# Comparative Effectiveness of Cell‐Based Versus Egg‐Based Influenza Vaccines in Prevention of Influenza Hospitalization During the 2022–2023 Season Among Adults 18–64 Years

**DOI:** 10.1111/irv.70025

**Published:** 2024-12-18

**Authors:** Emily Rayens, Jennifer H. Ku, Lina S. Sy, Lei Qian, Bradley K. Ackerson, Yi Luo, Julia E. Tubert, Gina S. Lee, Punam P. Modha, Yoonyoung Park, Tianyu Sun, Evan J. Anderson, Hung Fu Tseng

**Affiliations:** ^1^ Department of Research and Evaluation Kaiser Permanente Southern California Pasadena California USA; ^2^ Division of Clinical Development Moderna Inc Cambridge Massachusetts USA; ^3^ Division of Real‐World Evidence Analytics Moderna Inc Cambridge Massachusetts USA; ^4^ Department of Health Systems Science Kaiser Permanente Bernard J. Tyson School of Medicine Pasadena California USA

**Keywords:** epidemiology, Influenza, influenza vaccine, vaccine effectiveness

## Abstract

This retrospective cohort study evaluated the comparative vaccine effectiveness (cVE) of licensed standard‐dose cell‐based versus egg‐based influenza vaccines in preventing influenza hospitalization among adults 18–64 years during the 2022–2023 season. The cohort included eligible Kaiser Permanente Southern California members who received ≥ 1 dose of influenza vaccine (*n* = 848,334). The adjusted cVE against influenza hospitalization was −10.1% (95% CI: −49.8%, 37.8%) in the 18‐ to 49‐year‐old cohort. In the 50‐ to 64‐year‐old cohort, the adjusted cVE was 14.9% (−33.8%, 52.1%). Cell‐based and egg‐based influenza vaccines conferred comparable protection against influenza hospitalization in adults 18–64 years of age in the 2022–2023 season.

## 1 Introduction

Seasonal influenza causes significant clinical burden with an estimated 5 million cases of severe illness and up to 650,000 deaths worldwide [1, 2]. Vaccines represent the best option for prevention and control of influenza. In individuals younger than 65 years of age, the available influenza vaccines in the United States include inactivated influenza vaccine (IIV), recombinant influenza vaccine, and live attenuated influenza vaccine, with no preferential recommendation [3]. IIVs, including egg‐based and cell‐based vaccines, are widely produced and administered in the United States. Egg‐based IIVs represent the majority of the influenza vaccines available on the market. However, there are several limitations to this method of manufacturing, including the length of production, production capacity, and possibility of egg‐adapted changes occurring during production [4, 5].

Some observational studies have reported greater protection against influenza or influenza‐related medical encounters among adults who received cell‐based compared to egg‐based influenza vaccines [6]. However, there was considerable heterogeneity in these observations across study design, study setting, age group, and influenza season. The purpose of this analysis was to evaluate the comparative vaccine effectiveness (cVE) of the licensed cell‐based versus egg‐based standard‐dose (SD) influenza vaccines in preventing influenza‐related hospitalization among adults younger than 65 years during the 2022–2023 influenza season.

## 2 Methods

### 2.1 Study Setting

Kaiser Permanente Southern California (KPSC) is a large, integrated health care system with over 4.8 million members with demographic and socioeconomic characteristics representative of the population of Southern California [7]. Comprehensive patient information, including vaccinations, laboratory tests, diagnoses, and procedures, are captured in electronic health records (EHR). Vaccinations received outside of KPSC are imported into the EHR from external sources, including the California Immunization Registry, Care Everywhere, claims, and vaccination self‐reports with valid documentation. Care received outside KPSC is added to the EHR through claims reimbursement. This study was approved by the KPSC Institutional Review Board, with a waiver of informed consent.

### 2.2 Study Population

**FIGURE 1:**
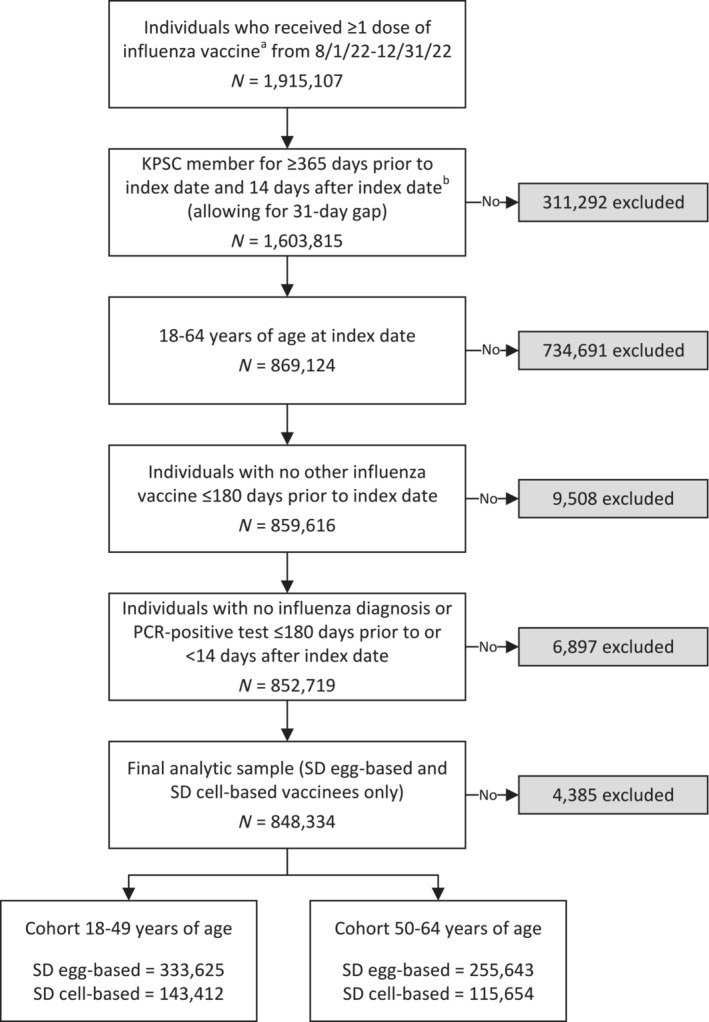
Flow diagram for the analytic cohort. KPSC = Kaiser Permanente Southern California; PCR = polymerase chain reaction; SD = standard dose. ^a^Influenza vaccines included SD egg‐based (CVX 150 and 158) and SD cell‐based (CVX 171 and 186) vaccines. ^b^Index date was defined as the date of the first dose of influenza vaccine received during the accrual period, based on the CVX codes above. Non‐index influenza vaccinations were not limited to the CVX codes above for identifying influenza vaccinations prior to index date (for exclusion criteria and covariates) and after index date (for censoring).

The study population included adults 18–64 years of age who received ≥ 1 dose of influenza vaccine during the accrual period 08/01/2022 to 12/31/2022, with follow‐up until 5/20/2023. The 2022–2023 season was characterized by early influenza activity that peaked in December 2022 [8]. The index date was defined as the date when the first influenza vaccination was administered during the accrual period. Individuals were required to have continuous KPSC membership (allowing for a 31‐day gap) for at least one year prior to and 14 days following the index date (Figure 1). Individuals were followed from 14 days after the index date until the outcome of interest (influenza‐related hospitalization), death, disenrollment (allowing for a 31‐day gap), receipt of another dose of influenza vaccine, or end of follow‐up, whichever came first. We excluded individuals who: (1) received an influenza vaccine ≤ 180 days prior to the index date; or (2) had evidence of influenza infection, including a listed diagnosis code or polymerase chain reaction (PCR)‐positive test ≤ 180 days prior to or within 14 days following the index date.

### 2.3 Exposure and Outcome

The exposure of interest was receipt of a pre‐specified licensed influenza vaccine during the accrual period identified using CVX (vaccine administered) codes (SD egg‐based = 150 and 158; SD cell‐based = 171 and 186). If an individual received > 1 influenza vaccine during the accrual period, follow‐up time related to the first vaccine was included, and the individual was censored at the time of receipt of the second vaccine. The primary outcome was PCR‐confirmed influenza‐related hospitalization, defined as a positive PCR test collected between 14 days prior to and 3 days following the inpatient admission date with an acute respiratory infection code [9].

### 2.4 Statistical Analysis

The study population was stratified into adults 18–49 and 50–64 years of age for statistical analysis. Baseline characteristics were described for each vaccine group; categorical variables were compared using the chi‐square test, and continuous variables were compared using the Kruskal–Wallis test. Stabilized inverse probability of treatment weighting (IPTW) was used to adjust for potential confounders [10]. Absolute standardized differences (ASD) were computed to assess the balance of covariates before and after weighting. An ASD < 0.10 was considered a negligible difference.

Weighted Cox proportional hazards regression models were used to estimate adjusted hazard ratios (aHR). Comparative vaccine effectiveness (cVE) (%) was calculated as (1 − aHR) × 100 when aHR ≤ 1, and ([1/aHR] − 1) × 100 when aHR > 1. All analyses were conducted using SAS (version 9.4; SAS Institute Inc, Cary, NC).

## 3 Results

Among the 1,915,107 individuals who received a dose of influenza vaccine during the accrual period, there were 477,037 individuals in the 18‐ to 49‐year‐old cohort and 371,297 in the 50‐ to 64‐year‐old cohort who met the eligibility criteria (Figure 1).

Patients were predominantly managed within the KPSC system at KPSC facilities where 795,169 (93.7%) of individuals included in this study received their influenza vaccines and where 105 (93.8%) of the 112 PCR‐confirmed influenza‐related hospitalizations were admitted. All patients with PCR‐confirmed influenza‐related hospitalizations had influenza A.

### 3.1 18‐ to 49‐Year‐Old Cohort

In the 18–49‐year‐old cohort overall, 61.1% were female, 44.6% were Hispanic, and the median age was 37 years (interquartile range [IQR] 29–43; Table 1). In this age group, 333,625 (69.9%) received the egg‐based vaccine. The egg‐based and cell‐based vaccinees were similar in demographic and clinical characteristics. The cell‐based vaccinees received their vaccines earlier compared to egg‐based vaccinees, at 69.4% and 65.8%, respectively, during August–October 2022 (Table S1). Baseline demographic characteristics, clinical characteristics, and healthcare utilization were well balanced (ASD < 0.1) after IPTW.

**TABLE 1:** Characteristics of influenza vaccine recipients 18–49 and 50–64 years of age by vaccine type (after inverse probability of treatment weighting).

*n* (%)	18–49 years				50–64 years			
	SD egg‐based	SD cell‐based	*p* value	ASD	SD egg‐based	SD cell‐based	*p* value	ASD
	*n* = 333,625	*n* = 143,412			*n* = 255,643	*n* = 115,654		
Demographic characteristics								
Age at index date, years			0.926	0.000			0.810	0.001
Mean (std dev)	35.6 (9.0)	35.6 (9.0)			57.4 (4.3)	57.4 (4.3)		
Median (Q1, Q3)	37 (29, 43)	37 (29, 43)			58 (54, 61)	58 (54, 61)		
Min, max	18, 49	18, 49			50, 64	50, 64		
Age at index date, years			0.423	0.004			0.770	0.001
18–29	89,842 (26.9)	38,808 (27.1)			N/A	N/A		
30–39	112,293 (33.7)	48,007 (33.5)			N/A	N/A		
40–49	131,490 (39.4)	56,597 (39.5)			N/A	N/A		
50–59	N/A	N/A			160,538 (62.8)	72,686 (62.8)		
60–64	N/A	N/A			95,105 (37.2)	42,968 (37.2)		
Sex			0.907	0.000			0.969	0.000
Female	203,787 (61.1)	87,626 (61.1)			141,495 (55.3)	64,005 (55.3)		
Male	129,838 (38.9)	55,786 (38.9)			114,148 (44.7)	51,649 (44.7)		
Race/ethnicity			0.723	0.005			0.815	0.004
Non‐Hispanic White	81,286 (24.4)	35,187 (24.5)			82,048 (32.1)	37,331 (32.3)		
Non‐Hispanic Black	15,141 (4.5)	6502 (4.5)			18,343 (7.2)	8293 (7.2)		
Hispanic	148,706 (44.6)	63,646 (44.4)			102,332 (40.0)	46,076 (39.8)		
Non‐Hispanic Asian	59,937 (18.0)	25,792 (18.0)			38,777 (15.2)	17,561 (15.2)		
Other/unknown	28,555 (8.6)	12,285 (8.6)			14,142 (5.5)	6393 (5.5)		
Medicaid	38,146 (11.4)	16,391 (11.4)	0.967	0.000	19,547 (7.6)	8849 (7.7)	0.815	0.004
Neighborhood median household income			0.890	0.003			0.885	0.004
< $40,000	4997 (1.5)	2148 (1.5)			3732 (1.5)	1686 (1.5)		
$40,000–$59,999	46,136 (13.8)	19,688 (13.7)			33,442 (13.1)	14,989 (13.0)		
$60,000–$79,999	74,178 (22.2)	31,827 (22.2)			54,416 (21.3)	24,608 (21.3)		
≥ $80,000	207,784 (62.3)	89,520 (62.4)			163,358 (63.9)	74,052 (64.0)		
Unknown	531 (0.2)	229 (0.2)			696 (0.3)	319 (0.3)		
Smoking^a^			0.976	0.001			0.995	0.000
No	263,105 (78.9)	113,062 (78.8)			193,424 (75.7)	87,490 (75.6)		
Yes	36,477 (10.9)	15,709 (11.0)			48,072 (18.8)	21,764 (18.8)		
Unknown	34,043 (10.2)	14,641 (10.2)			14,147 (5.5)	6400 (5.5)		
Clinical characteristics								
Body mass index^a^, kg/m^2^			0.993	0.002			1.000	0.001
< 18.5	4557 (1.4)	1971 (1.4)			1557 (0.6)	710 (0.6)		
18.5 to < 25	79,831 (23.9)	34,382 (24.0)			48,830 (19.1)	22,114 (19.1)		
25 to < 30	85,028 (25.5)	36,567 (25.5)			81,159 (31.7)	36,720 (31.7)		
≥ 30	116,957 (35.1)	50,207 (35.0)			102,373 (40.0)	46,280 (40.0)		
Unknown	47,252 (14.2)	20,284 (14.1)			21,724 (8.5)	9831 (8.5)		
Charlson comorbidity score^b^, ^c^			0.994	0.002			0.874	0.002
Mean (std dev)	0.3 (0.8)	0.3 (0.8)			0.74 (1.43)	0.7 (1.4)		
Median (Q1, Q3)	0 (0, 0)	0 (0, 0)			0 (0, 1)	0 (0, 1)		
Min, max	0, 16	0, 13			0, 17	0, 16		
Charlson comorbidity score^b^, ^c^			0.983	0.001			0.994	0.000
0	279,325 (83.7)	120,075 (83.7)			164,922 (64.5)	74,634 (64.5)		
1	39,214 (11.8)	16,839 (11.7)			48,449 (19.0)	21,907 (18.9)		
≥ 2	15,086 (4.5)	6499 (4.5)			42,272 (16.5)	19,113 (16.5)		
Frailty index^b^, ^d^			0.608	0.003			0.635	0.002
Mean (std dev)	0.1 (0.0)	0.1 (0.0)			0.1 (0.0)	0.1 (0.0)		
Median (Q1, Q3)	0.1 (0.1, 0.1)	0.1 (0.1, 0.1)			0.1 (0.1, 0.1)	0.1 (0.1, 0.1)		
Min, max	0.0, 0.4	0.1, 0.4			0.0, 0.4	0.0, 0.4		
Frailty index^b^, ^d^			0.983	0.001			1.000	0.000
Quartile 1	83,382 (25.0)	35,805 (25.0)			62,622 (24.5)	28,312 (24.5)		
Quartile 2	45,896 (13.8)	19,690 (13.7)			65,184 (25.5)	29,498 (25.5)		
Quartile 3	120,932 (36.2)	51,996 (36.3)			63,940 (25.0)	28,932 (25.0)		
Quartile 4, most frail	83,415 (25.0)	35,921 (25.0)			63,897 (25.0)	28,911 (25.0)		
Chronic diseases^b^								
Kidney disease	2468 (0.7)	1061 (0.7)	0.991	0.000	9224 (3.6)	4162 (3.6)	0.883	0.001
Heart disease	1558 (0.5)	674 (0.5)	0.882	0.001	6475 (2.5)	2926 (2.5)	0.961	0.000
Liver disease	7746 (2.3)	3341 (2.3)	0.870	0.001	12,162 (4.8)	5502 (4.8)	0.997	0.000
Diabetes	18,387 (5.5)	7890 (5.5)	0.891	0.000	51,824 (20.3)	23,403 (20.2)	0.797	0.001
Immunocompromised^e^	8878 (2.7)	3818 (2.7)	0.983	0.000	10,595 (4.1)	4797 (4.1)	0.963	0.000
Respiratory conditions^b^								
Chronic obstructive pulmonary disease, chronic bronchitis, or emphysema	3912 (1.2)	1691 (1.2)	0.854	0.001	6167 (2.4)	2804 (2.4)	0.819	0.001
Asthma	22,261 (6.7)	9557 (6.7)	0.913	0.000	16,762 (6.6)	7591 (6.6)	0.937	0.000
Healthcare utilization								
Number of outpatient and virtual visits^b^			0.995	0.001			0.999	0.001
0	3912 (1.2)	1691 (1.2)			10,513 (4.1)	4766 (4.1)		
1–4	22,261 (6.7)	9557 (6.7)			69,446 (27.2)	31,419 (27.2)		
5–10	3912 (1.2)	1691 (1.2)			84,261 (33.0)	38,130 (33.0)		
≥ 11	22,261 (6.7)	9557 (6.7)			91,422 (35.8)	41,338 (35.7)		
Number of emergency department visits^b^			0.979	0.001			0.998	0.000
0	284,111 (85.2)	122,120 (85.2)			215,586 (84.3)	97,529 (84.3)		
1	36,296 (10.9)	15,623 (10.9)			29,046 (11.4)	13,147 (11.4)		
≥ 2	13,218 (4.0)	5669 (4.0)			11,011 (4.3)	4977 (4.3)		
Number of hospitalizations^b^			0.987	0.001			0.986	0.001
0	317,205 (95.1)	136,338 (95.1)			245,846 (96.2)	111,210 (96.2)		
1	14,195 (4.3)	6116 (4.3)			7355 (2.9)	3339 (2.9)		
≥ 2	2226 (0.7)	958 (0.7)			2442 (1.0)	1105 (1.0)		
Preventive care^b^, ^f^	122,802 (36.8)	52,761 (36.8)	0.902	0.000	156,647 (61.3)	70,892 (61.3)	0.902	0.000
Receipt of influenza vaccine^g^	244,696 (73.3)	105,140 (73.3)	0.822	0.001	213,891 (83.7)	96,751 (83.7)	0.926	0.000
Receipt of COVID‐19 vaccine^b^	249,791 (74.9)	107,350 (74.9)	0.896	0.000	212,000 (82.9)	95,872 (82.9)	0.806	0.001
Concomitant vaccines^h^	61,917 (18.6)	26,582 (18.5)	0.849	0.001	69,080 (27.0)	31,303 (27.1)	0.780	0.001
Month of vaccination			0.982	0.002			0.986	0.002
August 2022	11,838 (3.5)	5088 (3.5)			10,551 (4.1)	4773 (4.1)		
September 2022	105,342 (31.6)	45,326 (31.6)			93,937 (36.7)	42,532 (36.8)		
October 2022	105,870 (31.7)	45,415 (31.7)			84,077 (32.9)	37,959 (32.8)		
November 2022	72,285 (21.7)	31,050 (21.7)			45,403 (17.8)	20,527 (17.7)		
December 2022	38,290 (11.5)	16,533 (11.5)			21,675 (8.5)	9863 (8.5)		

Abbreviations: ASD = absolute standardized difference; Q1 = quartile 1; Q3 = quartile 3; SD = standard dose; std dev = standard deviation.

^a^ Defined in the two years prior to index date.

^b^ Defined in the one year prior to index date.

^c^ Possible range: 0–29 [11].

^d^ Possible range: 0–1 [12].

^e^ HIV/AIDS, leukemia/lymphoma, congenital/other immunodeficiencies, asplenia/hyposplenia, hematopoietic stem cell transplant/solid organ transplant, and receipt of immunosuppressive medications.

^f^ Includes screenings, preventive physical exams, and wellness visits.

^g^ During previous influenza season (August 2021 to April 2022).

^h^ Administered on index date.

**FIGURE 2:**
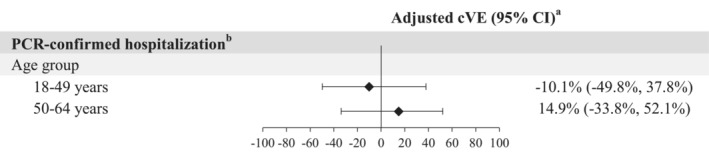
Adjusted cVE for the SD cell‐based influenza vaccine compared to the SD egg‐based vaccine. CI = confidence interval; cVE = comparative vaccine effectiveness (SD cell‐based vs. SD egg‐based); PCR = polymerase chain reaction; SD = standard dose. ^a^Weighted using stabilized inverse probability of treatment weights. When the hazard ratio or its 95% CI was > 1, the cVE (%) or its 95% CI was transformed as ([1/hazard ratio] − 1) × 100. ^b^PCR‐confirmed influenza‐related hospitalization (a positive PCR test collected between −14 and +3 days from the inpatient admission date) with an acute respiratory infection code [9].

The incidence rate (IR) of PCR‐confirmed influenza hospitalization per 1000 person‐years was 0.2 (95% confidence interval [CI]: 0.1–0.4) in those who received cell‐based vaccines and 0.2 (95% CI: 0.2–0.3) in those who received egg‐based vaccines (Table S2). The adjusted cVE was −10.1% (95% CI: −49.8%, 37.8%; Figure 2).

### 3.2 50‐ to 64‐Year‐Old Cohort

In the 50‐ to 64‐year‐old cohort overall, 55.4% were female, 40.1% were Hispanic, and the median age was 58 years (IQR 54–61; Table 1). In this age group, 255,643 (68.9%) received egg‐based vaccines. The egg‐based and cell‐based vaccinees were similar in most demographic and clinical characteristics, although cell‐based vaccinees were vaccinated slightly earlier than egg‐based vaccinees, at 75.1% and 73.1%, respectively, during August to October 2022 (Table S1). Baseline demographic characteristics, clinical characteristics, and healthcare utilization were well balanced (ASD < 0.1) after IPTW. The IR of PCR‐confirmed influenza hospitalization per 1000 person‐years was 0.2 (95% CI: 0.1–0.4) with cell‐based vaccination and 0.3 (95% CI: 0.2–0.4) with egg‐based vaccination (Table S2). The adjusted cVE was 14.9% (95% CI: −33.8%, 52.1%; Figure 2).

## 4 Discussion

In this study, we evaluated the cVE of the SD cell‐based influenza vaccine, compared to the SD egg‐based vaccine. We observed that cell‐based and egg‐based influenza vaccines conferred comparable protection against PCR‐confirmed influenza‐related hospitalization in adults 18–64 years of age in the 2022–2023 season.

While this analysis reports the cVE of egg‐ and cell‐based influenza vaccines, influenza vaccinations provided moderate protection against influenza‐related hospitalizations in adults aged 18–64 in the 2022–2023 season (VE: 23%; 95% CI: 4%–39%) [13]. Previous studies have found that cell‐based vaccines may provide moderately higher levels of protection against influenza‐related outcomes in adults younger than 64 years. In a meta‐analysis of IIVs, the overall relative VE of cell‐based vaccines in preventing medical encounters, compared to egg‐based vaccines, in persons 4–64 years of age was estimated to be 16.2% (95% CI: 7.6%–24.8%), 6.1% (95% CI: 4.9%–7.3%), 10.2% (95% CI: 6.3%–14.0%) for the 2017–2018, 2018–2019, and 2019–2020 seasons, respectively [6]. However, there was considerable heterogeneity across studies (*I* ^2^ = 79%), which may be related to variations in study design, study setting, age group, and influenza season.

All PCR‐confirmed influenza‐related hospitalizations were caused by influenza A, which was the predominant circulating virus subtype in the 2022–2023 influenza season [8]. Based on recommendations from WHO, the SD egg‐based and SD cell‐based influenza vaccines for the season consisted of comparable influenza A (H1N1 and H3N2) and influenza B (Victoria and Yamagata) strains [8]. Any differences in vaccine performance should not have been based on potential strain mismatch.

Our study has several notable strengths, including the use of a large cohort with comprehensive capture of demographic and clinical information by EHR. Second, confounding by indication was minimized as all individuals in the study were vaccinated. The distribution of covariates, except index month, was well balanced even before weighting (Table S1), and vaccine type administered was based on availability as opposed to patient choice. Finally, PCR results were used to increase the specificity of our outcome definition.

Nevertheless, our study has some limitations. Despite incentivizing KPSC members to receive vaccines within the KPSC system and the importing of external vaccination data, it is possible that a small number of influenza vaccines received outside the system could be missed. Misclassification of influenza‐related hospitalization is possible due to the reliance on molecular test results; however, molecular tests for influenza are highly sensitive [14]. Outcome misclassification was likely non‐differential by vaccine type. Although we adjusted for covariates, residual confounding may still exist but is likely to be minimal based on good balance of most measured confounders even prior to weighting. Our results address cVE against hospitalized influenza, but we did not assess less severe influenza‐associated medical encounters. Finally, these findings may not be generalizable to individuals who receive care in different types of health systems in the United States, in uninsured populations, or in other countries, but KPSC members are racially and ethnically diverse and generally representative of the Southern California population [15].

In summary, our results indicate that cell‐based and egg‐based influenza vaccines conferred comparable protection against PCR‐confirmed influenza‐related hospitalization in adults 18–64 years of age during the 2022–2023 season. Ongoing evaluations of existing influenza vaccines are crucial in guiding recommendations, revision of current vaccine candidates, and development of new influenza vaccines that balance protection, risk, and ease and speed of production.

## Author Contributions

**Emily Rayens:** writing – original draft, investigation. **Jennifer H. Ku:** investigation, conceptualization, methodology. **Lina S. Sy:** conceptualization, methodology, investigation, project administration. **Lei Qian:** conceptualization, methodology, formal analysis, investigation, data curation. **Bradley K. Ackerson:** conceptualization, methodology, investigation. **Yi Luo:** conceptualization, methodology, investigation, formal analysis, data curation. **Julia E. Tubert:** conceptualization, methodology, investigation, formal analysis, data curation. **Gina S. Lee:** investigation, project administration. **Punam P. Modha:** investigation, project administration. **Yoonyoung Park:** conceptualization, methodology, funding acquisition, project administration, investigation, supervision. **Tianyu Sun:** conceptualization, methodology, investigation. **Evan J. Anderson:** conceptualization, methodology, investigation, supervision. **Hung Fu Tseng:** conceptualization, methodology, investigation, funding acquisition, project administration, supervision.

## Ethics Statement

The study was approved by the Institutional Review Board (IRB) of Kaiser Permanente Southern California (KPSC) (reference number 13638).

## Conflicts of Interest

E.R., J.H.K., L.S.S., L.Q., B.K.A., Y.L., J.E.T., G.S.L., P.P.M., and H.F.T. are employees of Kaiser Permanente Southern California, which has been contracted by Moderna, Inc. to conduct this study. Y.P., T.S., and E.J.A. are employees of and shareholders in Moderna, Inc. E.R. received funding from GlaxoSmithKline unrelated to this manuscript. J.H.K. received funding from GlaxoSmithKline and Moderna unrelated to this manuscript. L.S.S. received funding from GlaxoSmithKline, Dynavax, and Moderna unrelated to this manuscript. L.Q. received funding from GlaxoSmithKline, Dynavax, and Moderna unrelated to this manuscript. B.K.A. received funding from GlaxoSmithKline, Dynavax, Genentech, and Moderna unrelated to this manuscript. Y.L. received funding from GlaxoSmithKline, Pfizer, and Moderna unrelated to this manuscript. J.E.T. received funding from GlaxoSmithKline and Moderna unrelated to this manuscript. G.S.L. received funding from GlaxoSmithKline and Moderna unrelated to this manuscript. P.P.M. received funding from GlaxoSmithKline unrelated to this manuscript. H.F.T. received funding from GlaxoSmithKline and Moderna unrelated to this manuscript; H.F.T. also served on advisory boards for Janssen Pharmaceuticals and Pfizer Inc.

### Peer Review

The peer review history for this article is available at https://www.webofscience.com/api/gateway/wos/peer‐review/10.1111/irv.70025.

## Supporting information


**Table S1.** Characteristics of influenza vaccine recipients 18–49 and 50–64 years of age by vaccine type (before inverse probability of treatment weighting).
**Table S2.** Incidence rates and comparative vaccine effectiveness of SD cell‐based influenza vaccines in preventing PCR‐confirmed influenza‐related hospitalization.

## Data Availability

Individual‐level data reported in this study involving human research participants are not publicly shared due to potentially identifying or sensitive patient information. Upon request to the corresponding author [ER], and subject to review and approval of an analysis proposal, Kaiser Permanente Southern California (KPSC) may provide the deidentified aggregate‐level data that support the findings of this study within 6 months. Anonymized data (deidentified data including participant data as applicable) that support the findings of this study may be made available from the investigative team in the following conditions: (1) agreement to collaborate with the study team on all publications, (2) provision of external funding for administrative and investigator time necessary for this collaboration, (3) demonstration that the external investigative team is qualified and has documented evidence of training for human subjects protections, and (4) agreement to abide by the terms outlined in data use agreements between institutions.
