# Supplementary material for: Comparative Effectiveness of Cell‐Based Versus Egg‐Based Influenza Vaccines in Prevention of Influenza Hospitalization During the 2022–2023 Season Among Adults 18–64 Years
Source: Influenza Other Respir Viruses. 2024 Dec 18;18(12):e70025. doi: 10.1111/irv.70025 (PMC11655916; doi:10.1111/irv.70025)
Supplement: Supplementary file 1 — Table S1. Characteristics of influenza vaccine recipients 18–49 and 50–64 years of age by vaccine type (before inverse probability of treatment weighting). Table S2. Incidence rates and comparative vaccine effectiveness of SD cell‐based influenza vaccines in preventing PCR‐confirmed influenza‐related hospitalization. [file IRV-18-e70025-s001.docx]

**Comparative effectiveness of cell-based versus egg-based influenza vaccines in prevention of influenza hospitalization during the 2022-2023 season among adults 18-64 years**

**Supporting Information**

Emily Rayens,^1^ Jennifer H. Ku,^1^ Lina S. Sy,^1^ Lei Qian,^1^ Bradley K. Ackerson,^1^ Yi Luo,^1^ Julia E. Tubert,^1^ Gina S. Lee,^1^ Punam P. Modha,^1^ Yoonyoung Park,^2^ Tianyu Sun,^2^ Evan J. Anderson,^2^ Hung Fu Tseng^1,3^

^1^Department of Research and Evaluation, Kaiser Permanente Southern California, Pasadena, CA 91101, USA
^2^Moderna Inc., Cambridge, MA 02142, USA

^3^Department of Health Systems Science, Kaiser Permanente Bernard J. Tyson School of Medicine, Pasadena, CA 91101, USA

**Keywords:** Influenza; influenza vaccine; vaccine effectiveness; epidemiology

**Running title**: Comparative effectiveness of influenza vaccines

**Corresponding author:**

Emily Rayens, PhD MPH

Kaiser Permanente Southern California, 100 S Los Robles Ave., Pasadena, CA 91101

Phone: (626) 564-3625

Email: [Emily.X.Rayens@kp.org](mailto:Emily.X.Rayens@kp.org)

ORCID: 0000-0003-3369-3475

Table of Contents

[**Table S1.** Characteristics of influenza vaccine recipients 18-49 and 50-64 years of age by vaccine type (before inverse probability of treatment weighting). 3](#_Toc167980005)

[**Table S2.** Incidence rates and comparative vaccine effectiveness of SD cell-based influenza vaccines in preventing PCR-confirmed influenza-related hospitalization. 5](#_Toc167980006)

# **Table S1.** Characteristics of influenza vaccine recipients 18-49 and 50-64 years of age by vaccine type (before inverse probability of treatment weighting).

| n (%) | **18-49 years** | | | | **50-64 years** | | | |
| --- | --- | --- | --- | --- | --- | --- | --- | --- |
|  | **SD Egg-based** | **SD Cell-based** | **p value** | **ASD** | **SD Egg-based** | **SD Cell-based** | **p value** | **ASD** |
|  | **n=333,625** | **n=143,412** |  |  | **n=255,643** | **n=115,654** |  |  |
| **Demographic characteristics** | |  |  |  |  |  |  |  |
| Age at index date, years | |  | 0.387 | 0.000 |  |  | <0.001 | 0.015 |
| Mean (std dev) | 35.6 (9.0) | 35.6 (9.1) |  |  | 57.4 (4.3) | 57.4 (4.3) |  |  |
| Median (Q1, Q3) | 37 (29, 43) | 37 (29, 43) |  |  | 58 (54, 61) | 58 (54, 61) |  |  |
| Min, max | 18, 49 | 18, 49 |  |  | 50, 64 | 50, 64 |  |  |
| Age at index date, years | | | <0.001 | 0.019 |  |  | <0.001 | 0.014 |
| 18-29 | 89556 (26.8) | 39168 (27.3) |  |  | N/A | N/A |  |  |
| 30-39 | 112969 (33.9) | 47289 (33.0) |  |  | N/A | N/A |  |  |
| 40-49 | 131100 (39.3) | 56955 (39.7) |  |  | N/A | N/A |  |  |
| 50-59 | N/A | N/A |  |  | 160068 (62.6) | 73209 (63.3) |  |  |
| 60-64 | N/A | N/A |  |  | 95575 (37.4) | 42445 (36.7) |  |  |
| Sex | |  | <0.001 | 0.020 |  |  | 0.269 | 0.004 |
| Female | 204770 (61.4) | 86607 (60.4) |  |  | 141340 (55.3) | 64168 (55.5) |  |  |
| Male | 128855 (38.6) | 56805 (39.6) |  |  | 114303 (44.7) | 51486 (44.5) |  |  |
| Race/Ethnicity | |  | <0.001 | 0.076 |  |  | <0.001 | 0.098 |
| Non-Hispanic White | 83576 (25.1) | 32519 (22.7) |  |  | 83965 (32.8) | 35056 (30.3) |  |  |
| Non-Hispanic Black | 15956 (4.8) | 5694 (4.0) |  |  | 19605 (7.7) | 7040 (6.1) |  |  |
| Hispanic | 147051 (44.1) | 65764 (45.9) |  |  | 100262 (39.2) | 48539 (42.0) |  |  |
| Non-Hispanic Asian | 58402 (17.5) | 27254 (19.0) |  |  | 37434 (14.6) | 18856 (16.3) |  |  |
| Other/Unknown | 28640 (8.6) | 12181 (8.5) |  |  | 14377 (5.6) | 6163 (5.3) |  |  |
| Medicaid | 37661 (11.3) | 16888 (11.8) | <0.001 | 0.015 | 19444 (7.6) | 8946 (7.7) | 0.170 | 0.005 |
| Neighborhood median household income | | | <0.001 | 0.040 |  |  | <0.001 | 0.051 |
| <$40,000 | 5330 (1.6) | 1815 (1.3) |  |  | 4055 (1.6) | 1367 (1.2) |  |  |
| $40,000-$59,999 | 46439 (13.9) | 19625 (13.7) |  |  | 33378 (13.1) | 15304 (13.2) |  |  |
| $60,000-$79,999 | 72926 (21.9) | 33176 (23.1) |  |  | 53189 (20.8) | 25848 (22.3) |  |  |
| ≥$80,000 | 208393 (62.5) | 88574 (61.8) |  |  | 164300 (64.3) | 72848 (63.0) |  |  |
| Unknown | 537 (0.2) | 222 (0.2) |  |  | 721 (0.3) | 287 (0.2) |  |  |
| Smoking^†^ | |  | <0.001 | 0.012 |  |  | <0.001 | 0.024 |
| No | 262637 (78.7) | 113590 (79.2) |  |  | 192687 (75.4) | 88252 (76.3) |  |  |
| Yes | 36700 (11.0) | 15441 (10.8) |  |  | 48466 (19.0) | 21344 (18.5) |  |  |
| Unknown | 34288 (10.3) | 14381 (10.0) |  |  | 14490 (5.7) | 6058 (5.2) |  |  |
| **Clinical characteristics** | | |  |  |  |  |  |  |
| Body Mass Index^†^, kg/m^2^ | | | <0.001 | 0.019 |  |  | <0.001 | 0.024 |
| <18.5 | 4604 (1.4) | 1904 (1.3) |  |  | 1623 (0.6) | 636 (0.5) |  |  |
| 18.5 - <25 | 80208 (24.0) | 33903 (23.6) |  |  | 48643 (19.0) | 22257 (19.2) |  |  |
| 25 - <30 | 84882 (25.4) | 36680 (25.6) |  |  | 80881 (31.6) | 36991 (32.0) |  |  |
| ≥30 | 116273 (34.9) | 51010 (35.6) |  |  | 102317 (40.0) | 46399 (40.1) |  |  |
| Unknown | 47658 (14.3) | 19915 (13.9) |  |  | 22179 (8.7) | 9371 (8.1) |  |  |
| Charlson comorbidity score^‡,§^ | | | 0.903 | 0.001 |  |  | <0.001 | 0.024 |
| Mean (std dev) | 0.3 (0.8) | 0.3 (0.8) |  |  | 0.8 (1.5) | 0.7 (1.4) |  |  |
| Median (Q1, Q3) | 0 (0, 0) | 0 (0, 0) |  |  | 0 (0, 1) | 0 (0, 1) |  |  |
| Min, max | 0, 16 | 0, 13 |  |  | 0, 17 | 0, 16 |  |  |
| Charlson comorbidity score^‡,§^ | | | 0.992 | 0.000 |  |  | <0.001 | 0.019 |
| 0 | 279304 (83.7) | 120082 (83.7) |  |  | 164587 (64.4) | 74930 (64.8) |  |  |
| 1 | 39235 (11.8) | 16849 (11.7) |  |  | 48231 (18.9) | 22144 (19.1) |  |  |
| ≥2 | 15086 (4.5) | 6481 (4.5) |  |  | 42825 (16.8) | 18580 (16.1) |  |  |
| Frailty index^‡,¶^ | |  | 0.096 | 0.007 |  |  | <0.001 | 0.023 |
| Mean (std dev) | 0.1 (0.0) | 0.1 (0.0) |  |  | 0.1 (0.0) | 0.1 (0.0) |  |  |
| Median (Q1, Q3) | 0.1 (0.1, 0.1) | 0.1 (0.1, 0.1) |  |  | 0.1 (0.1, 0.1) | 0.1 (0.1, 0.1) |  |  |
| Min, max | 0.0, 0.4 | 0.1, 0.4 |  |  | 0.0, 0.4 | 0.0, 0.4 |  |  |
| Frailty index^‡,¶^ | |  | 0.008 | 0.011 |  |  | <0.001 | 0.020 |
| Quartile 1 | 83319 (25.0) | 35930 (25.1) |  |  | 62155 (24.3) | 28802 (24.9) |  |  |
| Quartile 2 | 45559 (13.7) | 20079 (14.0) |  |  | 65149 (25.5) | 29518 (25.5) |  |  |
| Quartile 3 | 121207 (36.3) | 51701 (36.1) |  |  | 63818 (25.0) | 29050 (25.1) |  |  |
| Quartile 4, most frail | 83540 (25.0) | 35702 (24.9) |  |  | 64521 (25.2) | 28284 (24.5) |  |  |
| Chronic diseases^‡^ | |  |  |  |  |  |  |  |
| Kidney disease | 2523 (0.8) | 1007 (0.7) | 0.046 | 0.006 | 9547 (3.7) | 3856 (3.3) | <0.001 | 0.022 |
| Heart disease | 1597 (0.5) | 628 (0.4) | 0.058 | 0.006 | 6666 (2.6) | 2738 (2.4) | <0.001 | 0.015 |
| Liver disease | 7787 (2.3) | 3284 (2.3) | 0.353 | 0.003 | 12247 (4.8) | 5417 (4.7) | 0.157 | 0.005 |
| Diabetes | 18205 (5.5) | 8101 (5.6) | 0.008 | 0.008 | 51761 (20.2) | 23538 (20.4) | 0.462 | 0.003 |
| Immunocompromised^⁑^ | 8882 (2.7) | 3813 (2.7) | 0.945 | 0.000 | 10834 (4.2) | 4551 (3.9) | <0.001 | 0.015 |
| Respiratory conditions^‡^ | |  |  |  |  |  |  |  |
| Chronic obstructive pulmonary disease, chronic bronchitis, or emphysema | 4084 (1.2) | 1505 (1.0) | <0.001 | 0.017 | 6476 (2.5) | 2473 (2.1) | <0.001 | 0.026 |
| Asthma | 22328 (6.7) | 9511 (6.6) | 0.442 | 0.002 | 16792 (6.6) | 7544 (6.5) | 0.603 | 0.002 |
| **Healthcare utilization** | | |  |  |  |  |  |  |
| Number of outpatient and virtual visits^‡^ | | | <0.001 | 0.022 |  |  | <0.001 | 0.021 |
| 0 | 27224 (8.2) | 11132 (7.8) |  |  | 10795 (4.2) | 4472 (3.9) |  |  |
| 1-4 | 120843 (36.2) | 52902 (36.9) |  |  | 69051 (27.0) | 31811 (27.5) |  |  |
| 5-10 | 91709 (27.5) | 39888 (27.8) |  |  | 84081 (32.9) | 38294 (33.1) |  |  |
| ≥11 | 93849 (28.1) | 39490 (27.5) |  |  | 91716 (35.9) | 41077 (35.5) |  |  |
| Number of Emergency Department visits^‡^ | | | <0.001 | 0.015 |  |  | <0.001 | 0.023 |
| 0 | 283595 (85.0) | 122640 (85.5) |  |  | 214987 (84.1) | 98130 (84.8) |  |  |
| 1 | 36642 (11.0) | 15247 (10.6) |  |  | 29351 (11.5) | 12834 (11.1) |  |  |
| ≥2 | 13388 (4.0) | 5525 (3.9) |  |  | 11305 (4.4) | 4690 (4.1) |  |  |
| Number of hospitalizations^‡^ | | | <0.001 | 0.027 |  |  | <0.001 | 0.032 |
| 0 | 316626 (94.9) | 136939 (95.5) |  |  | 245390 (96.0) | 111681 (96.6) |  |  |
| 1 | 14687 (4.4) | 5603 (3.9) |  |  | 7638 (3.0) | 3041 (2.6) |  |  |
| ≥2 | 2312 (0.7) | 870 (0.6) |  |  | 2615 (1.0) | 932 (0.8) |  |  |
| Preventive care^‡,x^ | 122370 (36.7) | 53233 (37.1) | 0.004 | 0.009 | 156061 (61.0) | 71431 (61.8) | <0.001 | 0.015 |
| Receipt of influenza vaccine^#^ | 243026 (72.8) | 106873 (74.5) | <0.001 | 0.038 | 212908 (83.3) | 97753 (84.5) | <0.001 | 0.034 |
| Receipt of COVID-19 vaccine^‡^ | 248206 (74.4) | 108968 (76.0) | <0.001 | 0.037 | 211439 (82.7) | 96491 (83.4) | <0.001 | 0.019 |
| Concomitant vaccines^+^ | 62637 (18.8) | 25894 (18.1) | <0.001 | 0.019 | 69192 (27.1) | 31101 (26.9) | 0.268 | 0.004 |
| Month of vaccination | |  | <0.001 | 0.163 |  |  | <0.001 | 0.136 |
| August 2022 | 10672 (3.2) | 6253 (4.4) |  |  | 9718 (3.8) | 5604 (4.8) |  |  |
| September 2022 | 106192 (31.8) | 44389 (31.0) |  |  | 95446 (37.3) | 40942 (35.4) |  |  |
| October 2022 | 102693 (30.8) | 48757 (34.0) |  |  | 81783 (32.0) | 40397 (34.9) |  |  |
| November 2022 | 71298 (21.4) | 32065 (22.4) |  |  | 44623 (17.5) | 21326 (18.4) |  |  |
| December 2022 | 42770 (12.8) | 11948 (8.3) |  |  | 24073 (9.4) | 7385 (6.4) |  |  |

SD=standard dose; ASD=absolute standardized difference; std dev=standard deviation; Q1=quartile 1; Q3=quartile 3.

^†^ Defined in the two years prior to index date.

^‡^ Defined in the one year prior to index date.

^§^ Possible range: 0-29.^14^

^¶^ Possible range: 0-1.^15^

^⁑^ HIV/AIDS, leukemia/lymphoma, congenital/other immunodeficiencies, asplenia/hyposplenia, hematopoietic stem cell transplant/solid organ transplant, and receipt of immunosuppressive medications.

^×^Includes screenings, preventive physical exams, and wellness visits

^#^ During previous influenza season (August 2021– April 2022).

^+^ Administered on index date.

# **Table S2.** Incidence rates and comparative vaccine effectiveness of SD cell-based influenza vaccines in preventing PCR-confirmed influenza-related hospitalization.

|  | **SD Cell-based** | | | | **SD Egg-based** | | | |  |  |
| --- | --- | --- | --- | --- | --- | --- | --- | --- | --- | --- |
| **Age group** | N | Number of cases | Number of person years | Incidence per 1,000 person-years  (95% CI) | N | Number of cases | Number of person years | Incidence per 1,000 person-years (95% CI) | Adjusted^†^ hazard ratio (95% CI) | Adjusted^†^ cVE  (%)  (95% CI) |
| 18-49 years | 143,412 | 17 | 76,816.9 | 0.2 (0.1-0.4) | 333,625 | 36 | 176,007.4 | 0.2 (0.2-0.3) | 1.1 (0.6, 2.0) | -10.1% (-49.8, 37.8) |
| 50-64 years | 115,654 | 15 | 63,784.6 | 0.2 (0.1-0.4) | 255,643 | 44 | 139,668.4 | 0.3 (0.2-0.4) | 0.9 (0.5, 1.5) | 14.9 (-33.8, 52.1) |

SD=standard dose; CI=confidence interval; cVE=comparative vaccine effectiveness; PCR=polymerase chain reaction.

^†^ Weighted using stabilized inverse probability of treatment weights. When the hazard ratio or its 95% CI was >1, the cVE or its 95% CI was transformed as ([1/hazard ratio] – 1) × 100.
